# Preliminary results of the Phase IIa trial of a folate binding protein (FBP) adjuvant cancer vaccine (E39+GM-CSF) in ovarian and endometrial cancer patients to prevent recurrence

**DOI:** 10.1186/2051-1426-2-S3-P74

**Published:** 2014-11-06

**Authors:** Julia Greene, Erika Schneble, John Berry, Alfred Trappey, Timothy Vreeland, Guy Clifton, William McGuire, George Maxwell, Sathibalan Ponniah, George Peoples

**Affiliations:** 1 San Antonio Military Medical Center, Houston, TX, USA; 2 MD Anderson Cancer Center, Houston, TX, USA; 3 Inova Fairfax Hospital, Falls Church, VA, USA; 4 Uniformed Services University of the Health Sciences, Bethesda, MD, USA; 5 CDVP, USUHS, Bethesda, MD, USA

## Background

FBP (aka Folate Receptor-a) is an immunogenic protein that is over-expressed in breast, endometrial (EC) and ovarian cancer (OC). FBP expression in malignant cells is 20-80 fold higher compared to the limited distribution in normal cells. We have completed enrollment of a Phase IIa clinical trial with E39, an HLA-A2 restricted, FBP peptide vaccine + GM-CSF. The vaccine is administered in the adjuvant setting to prevent recurrences in high-risk, EC and OC patients (pts) rendered clinically disease-free with standard-of-care therapy. Here, we summarize preliminary toxicity (tox), immunologic and clinical response to vaccination.

## Methods

HLA-A2+ pts are enrolled into the vaccine group (VG) while HLA-A2- pts are being followed prospectively as an untreated control group (CG). Six monthly intradermal inoculations (V1-V6) of either100mcg, 500mcg, or 1000mcg of E39 + 250 mcg GM-CSF are administered during the primary vaccine series (PVS). *In vivo *immunologic responses are assessed by both local reactions (LR) after each inoculation and delayed hypersensitivity (DTH) reactions measured at baseline (R0) and after the PVS (R6). Recurrences are determined clinically. Data are means compared with a paired, t-test or Chi-square/Fisher Exact test as appropriate.

## Results

47 pts have enrolled; 25 in the VG and 22 in the CG. There are no significant differences in age, grade, stage, or nodal status between groups (all P > 0.20*)*. Overall, the vaccine was well-tolerated (max local tox: 96% Grade (Gr) 1, 4% Gr 2; max systemic tox: 56.5% Gr 1, 26.1% Gr 2, 4.3% Gr 3). The LR significantly increased from V1 to V2 (45.6mm ± 5.5 v 71.9mm ± 6.6, p < 0.01), and then plateaued between V3 and V6 (93.4mm ± 8.3 v 102.4mm ± 10.6, p = 0.51). With 14 patients having completed the PVS, DTH trended toward an increase from R0 to R6 (7.15mm ± 1.7 v 15.8mm ± 4.2, p = 0.07). After a median follow-up of 13 months, there have been 11/22 (50%) recurrences in the CG compared to 8/25 (32%) recurrences in the VG (p = 0.25).

## Conclusions

Current results from this Phase IIa trial indicate the E39 vaccine is well-tolerated and elicits a strong *in vivo *immune response. Preliminarily, the vaccine appears to reduce the risk of recurrence in adjuvant treated EC and OC patients. In contrast to other FBP-directed cytotoxic agents, the E39 vaccine holds the promise of a sustained immunologic response and memory against FBP-expressing recurrences with minimal toxicity.
